# Increased Pro Th1 And Th17 Transcriptional Activity In Patients With Severe COVID-19

**DOI:** 10.7150/ijms.80498

**Published:** 2023-02-21

**Authors:** Marina Jovanovic, Sofija Sekulic, Miodrag Jocic, Milena Jurisevic, Nevena Gajovic, Marina Jovanovic, Nebojsa Arsenijevic, Milan Jovanovic, Milan Mijailovic, Milos Milosavljevic, Ivan Jovanovic

**Affiliations:** 1 University of Kragujevac, Serbia, Faculty of Medical Sciences, Department of Otorhinolaringology; 2 University of Kragujevac, Serbia, Faculty of Medical Sciences, Department of Infectious Disease; 3 Military Medical Academy, Belgrade, Serbia, Institute for Transfusiology and Haemobiology; 4 University of Kragujevac, Serbia, Faculty of Medical Sciences, Department of Clinical Pharmacy; 5 University of Kragujevac, Serbia, Faculty of Medical Sciences, Center for Molecular Medicine and Stem Cell Research; 6 University of Kragujevac, Serbia, Faculty of Medical Sciences, Department of Internal medicine; 7 Military Medical Academy, Belgrade, Serbia, Department of Abdominal Surgery; 8 University of Kragujevac, Serbia, Faculty of Medical Sciences, Department of Radiology

**Keywords:** CD4^+^ T cells, Th1, Th17, Tbet, RORγT, COVID-19 severity

## Abstract

**Background**: COVID-19 is known to disrupt immune response and induce hyperinflammation that could potentially induce fatal outcome of the disease. Until now, it is known that interplay among cytokines is rather important for clinical presentation and outcome of COVID-19. The aim of this study was to determine transcriptional activity and functional phenotype of T cells and the relationship between pro- and anti-inflammatory cytokines and clinical parameters of COVID-19 severity.

**Methods**: All recruited patients met criteria for COVID-19 are were divided in four groups according to disease severity. Serum levels of IL-12, IFN-γ, IL-17 and IL-23 were measured, and flow cytometry analysis of T cells from peripheral blood was performed.

**Results**: Significant elevation of IL-12, IFN-γ, IL-17 and IL-23 in stage IV of the disease has been revealed. Further, strong intercorrelation between IL-12, IFN-γ, IL-17 and IL-23 was also found in stage IV of the disease, marking augmented Th1 and Th17 response. Analyses of T cells subsets indicate a noticeable phenotype change. CD4^+^, but not CD8^+^ T cells expressed increased transcriptional activity through increased expression of Tbet and RORγT, accompanied with increased percentage of IFN-γ and IL-17 producing T cells.

**Conclusion**: Our results pose a novel hypothesis of the underlying mechanism behind deteriorating immune response in severe cases of COVID-19.

## Background

COVID-19 disease caused by a novel coronavirus (SARS-CoV-2), which started in Wuhan, China on December 2019, is spreading rapidly throughout the world 1. COVID-19 patients showed various clinical and laboratory findings, which can be from asymptomatic, through mild and moderate form to acute respiratory distress syndrome (ARDS) and respiratory insufficiency 2. COVID-19 patients also have increased levels of plasma pro-inflammatory cytokines such as interleukin (IL)-1β, IL-1RA, IL-6, IL-12, IFN-γ, IL-8, IL-10 with Th1/Th17 cells appearing as the source 3. It has been acknowledged that cytokines of Th1 response, IFN-γ and IL-12, alongside with IL-1 might be one of first cytokines to induce anti-viral immune response 4. During SARS-CoV-2 infection, IFN-γ secretion is increased and could induce potentially hazardous immune response 5. The study by *Liu et al.* emphasized the role of IFN-γ in lung damage 6. IL-12, on the other hand, might stimulate further production of IFN-γ in a positive feedback mechanism 7. Study by *Chen et al.* underlined that IL-12 is highly elevated in serum of COVID-19 patients 8. It is highly likely that, when SARS-CoV-2 infection occurs, both of these cytokines IFN-γ and IL-12, act synergistically, and by positive feedback mechanism, might induce damage to various organs 9. In a recently published study, activation of the Th17 pathway was shown in patients with COVID-19 10. One of the main cytokines produced by Th17- lymphocytes is IL-17. It is known that the overproduction of IL-17 stimulates the T-cell response and increases the production of other inflammatory mediators IL-1β, IL-6, TNF-α, granulocytes and granulocyte-macrophage colony-stimulating factors (G-CSF, GM-CSF), and various chemokines 11. The activation of Th17-lymphocytes and the production of IL-17 increases of the recruitment of neutrophils and prevents their apoptosis, which ultimately increases the damage to the lung tissue, and contributes to the development of pulmonary edema 11, 12. Interleukin-17 (IL-17) is a cytokine that is often involved in a proinflammatory response in the cytokine storm of viral infections. IL-17 has been suggested to be involved in the development of endothelial dysfunction and thrombophilia in COVID-19 13. In addition, Th17 cells also produce Interleukin-23 (IL-23), which plays a protective/anti-inflammatory role, and it is dysregulated in several proinflammatory conditions 14. Interestingly, the role of immunosuppressive cytokines, especially IL-10, may also contribute to COVID-19 induced damage to the lungs in later stages of the disease 9.

Due to the exact mechanisms of respiratory failure and impeding proinflammatory cascade in COVID-19 are still challenging, we hypothesized that severe form of COVID-19 (IV stage) has stronger Th17 cell proinflammatory response. Also, we performed this study to determine the association between pro-, anti-inflammatory and innate immunity cytokines disease severity, alongside with clinical and biochemical features of COVID-19 patients.

## Material and Methods

### Patients

In this study, 280 patients with confirmed COVID-19 were involved and examined. COVID-19 was verified by real-time reverse transcription-PCR (RT-PCR) analysis. All enrolled patients were divided into four groups depending on disease severity according to following criteria:

1) Mild group involved of 70 patients with fever (37-38°C), fatigue, pharyngalgia, nausea and vomiting, headache, dry irritating cough, dyspnea, SaO_2_ 92-100%, pO_2_ 8.5 kPa- 13.3 kPa, with normal or attenuated respiratory noise, with normal or interstitial thickening in CXR lung findings (CXR I, II);

2) Moderate group involved 70 patients with fever (38-39°C), fatigue, pharyngalgia, nausea and vomiting, headache, dry irritating cough, dyspnea, SaO_2_ 83%-91%, pO_2_ 7.1 kPa-8.4 kPa, with normal or attenuated/sharpened respiratory noise, and audible cracks in the lower segments of the lungs, with interstitial thickening and/or unifocal consolidation in CXR lung findings (III);

3) Severe group involved 70 patients with fever of 39-39,5°C, frequent dry irritating cough, dyspnea, fatigue, myalgia, nausea and vomiting, headache, chest pain, SaO_2_ 75%-82%, pO_2_ 5.6 kPa-7.1 kPa with required high-frequency ventilation (HFV), non-invasive mechanical ventilation (Non-Invasive Ventilation, NIV), or invasive mechanical ventilation, with auscultatory attenuated/inaudible respiratory noise or with audible whistling or cracks diffusely, with multifocal consolidation, diffuse alveolar changes, ARDS in CXR lung findings (CRX IV):

4) Critical group involved 70 patients with fever of 39,5-40°C, persistent dry irritating cough, dyspnea, fatigue, myalgia, nausea and vomiting, headache, chest pain, SaO_2_ ≤ 68%-74%, pO2 ≤ 4.2 kPa-5.5 with required high-frequency ventilation (HFV), non-invasive mechanical ventilation (Non-Invasive Ventilation, NIV), or invasive mechanical ventilation, with auscultatory attenuated or inaudible respiratory noise with audible whistling or cracks diffusely, with multifocal consolidation and/or diffuse alveolar changes, ARDS in CXR lung findings (CRX V).

None of enrolled patients were using antibiotics, corticosteroids, immunosuppressive agents, aminosalicylates or any kind of biological therapy for at least 2 months before the start of the study.

### Data Collection

All potentially infected COVID-19 patients had nasopharyngeal swab samples taken and then sent to authorized laboratories designated to detect the presence of SARS-CoV-2 virus, as previously described 15.

All patients had venous puncture blood sampling for the purposes of: D-dimer analyses, blood cell counting, immunoassays and biochemical parameters in plasma (urea, creatinine, glycemia, CRP, CK, PCT, AST, ALT, LDH, ALB, DBIL, TBIL, iron, ferritin, potassium and sodium). The analyses were conducted according to standard methods using the Beckman Coulter AU 400 Unicel DXC 800 Synchron Clinical System, in the Central Biochemical Laboratory of UKC Kragujevac. The patients also had arterial blood gases (SaO_2_, pO_2_, pCO_2_) measured multiple times during the day by ion selective electrode on the automaton. CXR Digital portable anteroposterior (AP) technique was used on all patients in the study to assess lung damage. In accordance with a V-point scoring scale, radiographic characteristics of patients were described as: I - normal; II - patchy atelectasis and/or hyperinflation and/or bronchial wall thickening; III - focal consolidation; IV - multifocal consolidation; and V - diffuse alveolar changes 16.

### Measurement of Cytokine Levels in Sera

As described in our previous study, all COVID-19 patients had their blood samples collected at 8:00 am 15. The separated sera was stored at -80 °C before use. In the sera samples we measured the concentrations of IL-12, IL-17, IL-23, IFN-γ and TGF-β using commercially available ELISA tests in accordance with manufacturer's instructions (R&D Systems, Minneapolis, Minn, USA).

### Immunophenotyping of basic lymphoid subpopulations by flow cytofluorometry

Fluorochrome- labeled mAbs specific for human CD3, CD4, CD8, CD56, Foxp3, Tbet, RORγt, IFN-γ, IL-17 or isotype-matched controls (BD Pharmingen, NJ / Invitrogen, / BioLegend / eBiosciences Carlsbad, CA) were used. For intracellular staining, cells were stimulated with phorbol 12-myristate 13-acetate (50 ng / mL, Sigma-Aldrich), ionomycin (500 ng / mL, Sigma-Aldrich) and GolgyStop (BD Pharmingen, NJ). Flow cytometry was performed on a FACS Calibur flow cytometer (BD Biosciences, San Jose, CA) and data analyzed using FlowJo (Tree Star).

### Statistical analysis

IBM SPSS Statistical Analysis Software (version 23.0) was used for all completed data analyses. The significance tests used for suitable purposes were Chi-square test, Student's t-test or Mann-Whitney U test. Data were presented as mean ± standard error of mean (SEM). The Pearson's or Spearman's correlation evaluated the possible relation between the variables. The strength of correlation was defined as negative or positive weak (-0.3 to -0.1 or 0.1 to 0.3), moderate (-0.5 to -0.3 or 0.3 to 0.5), or strong (-1.0 to -0.5 or 0.5 to 1). Receiver operating characteristic (ROC) curve analysis was used to determine the optimal limit values for IL-12, IFN-γ, IL-17 and IL-23 levels to detect the severity of COVID-19. The statistical significance was set at p < 0.05.

### Ethical statement

This study was performed in Covid Center of the University of Clinical Center Kragujevac (Ethical Approval Number 01/20-406) and in Center for Molecular Medicine and Stem Cell Research of Faculty of Medical Sciences, University of Kragujevac, Serbia (Ethical Approval Number 01-6776). All examined patients signed informed consent. All research procedures were conducted in compliance with the Helsinki Declaration and the Principle of Good Clinical Practice.

## Results

All recruited COVID-19 patients were divided into four groups, based on disease severity. The analysis of the data did not show any significant difference regarding the distribution of gender and age accross four stages of the disease. Also, there were not significant dissimilarities among patients when it comes to history of cardiovascular disease, diabetes or any other chronic illness. All laboratory findings (D-dimer analyses, blood cell counting, urea, creatinine, glycemia, CRP, CK, PCT, AST, ALT, LDH, ALB, DBIL, TBIL, iron, ferritin, potassium and sodium) were higher with increasing disease severity (data not shown).

### Patients with more severe stage of COVID-19 had increased systemic values of Th1/Th17 cytokines

**Figure 1:**
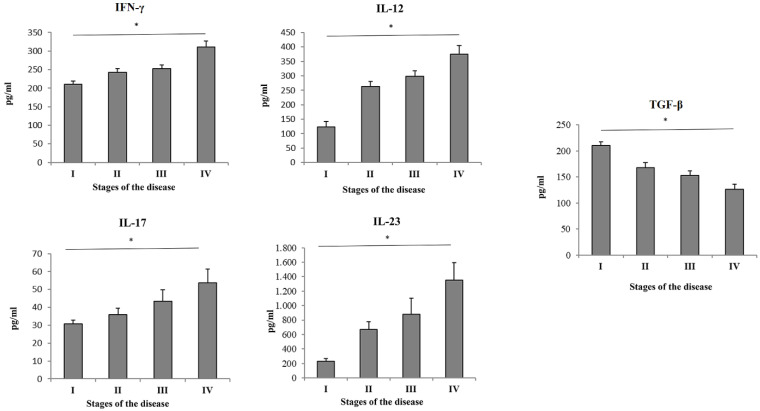
Serum values of pro- and anti-inflammatory cytokines. Based on the disease severity, all COVID-19 patients were divided into four groups: I, II, III and IV. Systemic levels of IL-12, IL-17, IL-23, IFN-γ and TGF-β were measured by ELISA. Statistical significance was tested by Mann-Whitney Rank Sum test.

Serum values of cytokines of interest were measured in all 4 groups of COVID-19 patients. High significant difference of systemic concentration of cytokines and IL-12 (p = 0. 001), IFN-γ (p = 0. 016), IL-23 (p = 0. 005) and IL-17 (p=0,008) have been found in COVID-19 patients in stage IV compared to patients in stage I, II and III (Figure 1). The value of TGF-β (p = 0. 030) was significantly lower in patients with advanced stage of COVID-19 (Figure 1).

### Strong inter-correlation between proinflammatory cytokines in the severe stage of COVID-19

In order to determine the relationship between proinflammatory cytokines in all stages of COVID-19, their inter-correlation has been analyzed. In patients in disease stages I, II and III, weaker correlations were detected compared to stage IV. In patients with stage IV of COVID-19, moderate positive correlation was detected between the serum values of IFN-γ and IL-12 (p = 0.005), IFN-γ and IL-17 (p = 0.014) (Table 1). Analysis revealed a strong positive correlation between IL-23 and IFN-γ (p = 0.000); IL-23 and IL-12 (p = 0.000) and IL-23 and IL-17 (p = 0.001) (Table 1). Moreover, analysis revealed no correlation between the serum values of IL-12 and IL-17 (p = 0.308) in patients with stage IV of COVID-19 (Table 1). Furthermore, FACS analysis revealed significant positive correlation between CD4^+^RORγt and CD3^+^IL-17^+^ cells (p = 0. 009) and high significant positive correlation between CD4^+^IL10^+^ and CD4^+^Foxp3^+^ cells (p = 0. 000) in stage IV patients compared to patients in stage I (Table 2).

### Correlation between cytokines (IL-17, IFN-γ, IL-12, IL-23) and clinical parameters in patients with COVID-19

Further, we calculated the correlation between serum level of cytokines of interest and clinical parameters of COVID-19. When it comes to IL-12, the analysis revealed high positive correlation between serum level of IL-12 and BILD (r = 0,229, p = 0.001), CRP (r = 0,224, p = 0.001), and CXR findings (r = 0,469, p = 0.013); and moderate positive correlation between serum IL-12 and the percentage of Neutrophils (r = 0,152, p = 0.028), Urea (r = 0,188, p = 0.006), LDH (r = 0,161, p = 0.027), CK (r = 0,154, p = 0.025), Na (r = 0,165, p = 0.016), D dimer (r = 0,172, p = 0.012), and PCT (r = 0,199, p = 0.003); but also a negative correlation between serum level of IL-12 and the percentage of Monocyte (r = - 0,234, p = 0.001), SAT (r = - 0,354, p = 0.000), Fe (r = - 0,214, p = 0.005), pO2 (r = - 0,369, p = 0.000) (Table 3). When it comes to IFN-γ, there was high positive correlation between serum level IFN-γ and age (r = 0,251, p = 0.000), and CXR findings (r = 0,378, p = 0.000); moderate positive correlation between serum level of IFN-γ and Urea (r = 0,189, p = 0.006), LDH (r = 0,225, p = 0.002), and PCT (r = 0,180, p = 0.009); and negative correlation between serum level of IFN-γ and SAT (r = - 0,227, p = 0.001), Albumins level (r = - 0,165, p = 0.018), and pO2 (r = - 0,269, p = 0.000), respectively (Table 3). When it comes to IL-17, the analysis revealed moderate positive correlation between serum level of IL-17 and CXR findings (r = 0,173, p = 0.013); and negative correlation between serum level of IL-17 and pH (r = - 0,169, p = 0.017) and K (r = - 0,133, p = 0.057) (Table 3). Also, serum level of IL-23 was in a moderate positive correlation with CK (r = 0,205, p = 0.003), and CRP (r = 0,165, p = 0.016); and in negative correlation with SAT (r = - 0,149, p = 0.030), and pO2 (r = - 0,226, p = 0.001) (Table 3). The results also have shown high positive correlation between disease severity (groups I to IV) and level of all cytokines: IL-12 (r = 0,463, p = 0.000), IL-23 (r = 0,371, p = 0.000), IFN-γ (r = 0,360, p = 0.000), and IL-17 (r = 0,178, p = 0.010), respectively (Table 3).

Parameters pO2 and SaO2 were also in significant negative association with COVID-19 severity (Table 3). Our results also illustrated that the serum levels of IL-12, IL-23, and IFN-γ are risk factors associated with COVID-19 severity.

### ROC curves analyses of serum level of IL-17, IFN-γ, IL-12 and IL-23 in COVID-19 patients

**Figure 2:**
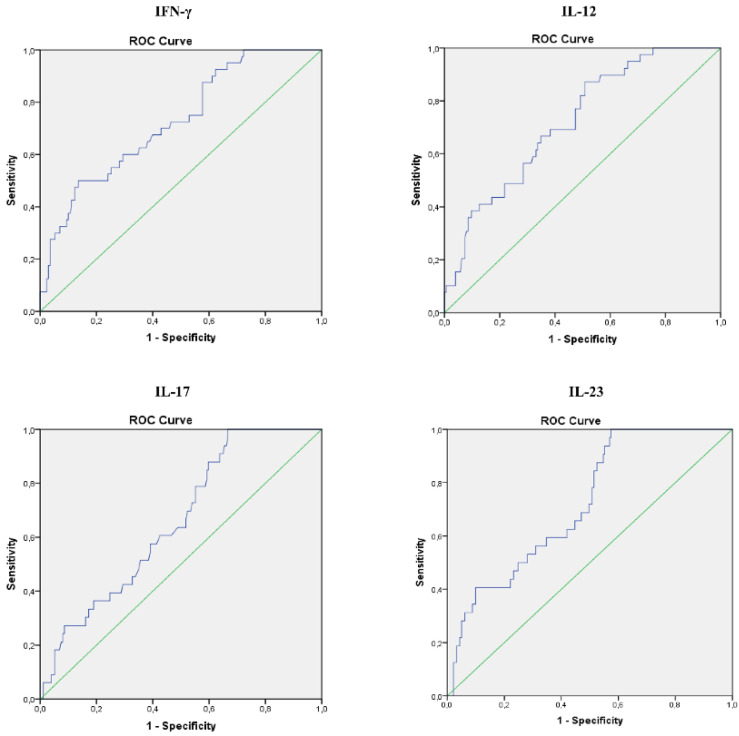
Specificity and sensitivity of serum IL-17, IFN-γ, IL-12 and IL-23, as possible markers for stage of IV COVID 19. Strength of correlation was determined using Spearman's test.

Analysis of Receiver Operating Characteristic (ROC) curves of serum IL-17, IFN-γ, IL-12 and IL-23 for different stages of COVID-19 revealed that values of all four cytokines could predict disease severity (Figure 5). Analysis showed that IL-17 can be an important marker for critical stage of COVID-19, with the optimum cut-off point 24,69 pg/ml (AUC 0.658; Sensitivity 84.8%; Specificity 40,8%; 95% CI 0.568-0.747; p = 0.004), as well as IFN-γ, with the optimum cut-off point 293,31 pg/ml (AUC 0.725; Sensitivity 50,0%; Specificity 81,2%; 95% CI 0.640-0.810; p = 0.001), IL-12, with the optimum cut-off point 215,16 pg/ml (AUC 0.722; Sensitivity 89,7%; Specificity 41,7%; 95% CI 0.641-0.803; p = 0.001), and IL-23 with the optimum cut-off point 230,75 pg/ml (AUC 0.721; Sensitivity 87,5%; Specificity 45,3%; 95% CI 0.636-0.806; p = 0.043) (Figure 2).

### Variations of percentage T cell subsets fractions across stages of COVID-19

**Figure 3:**
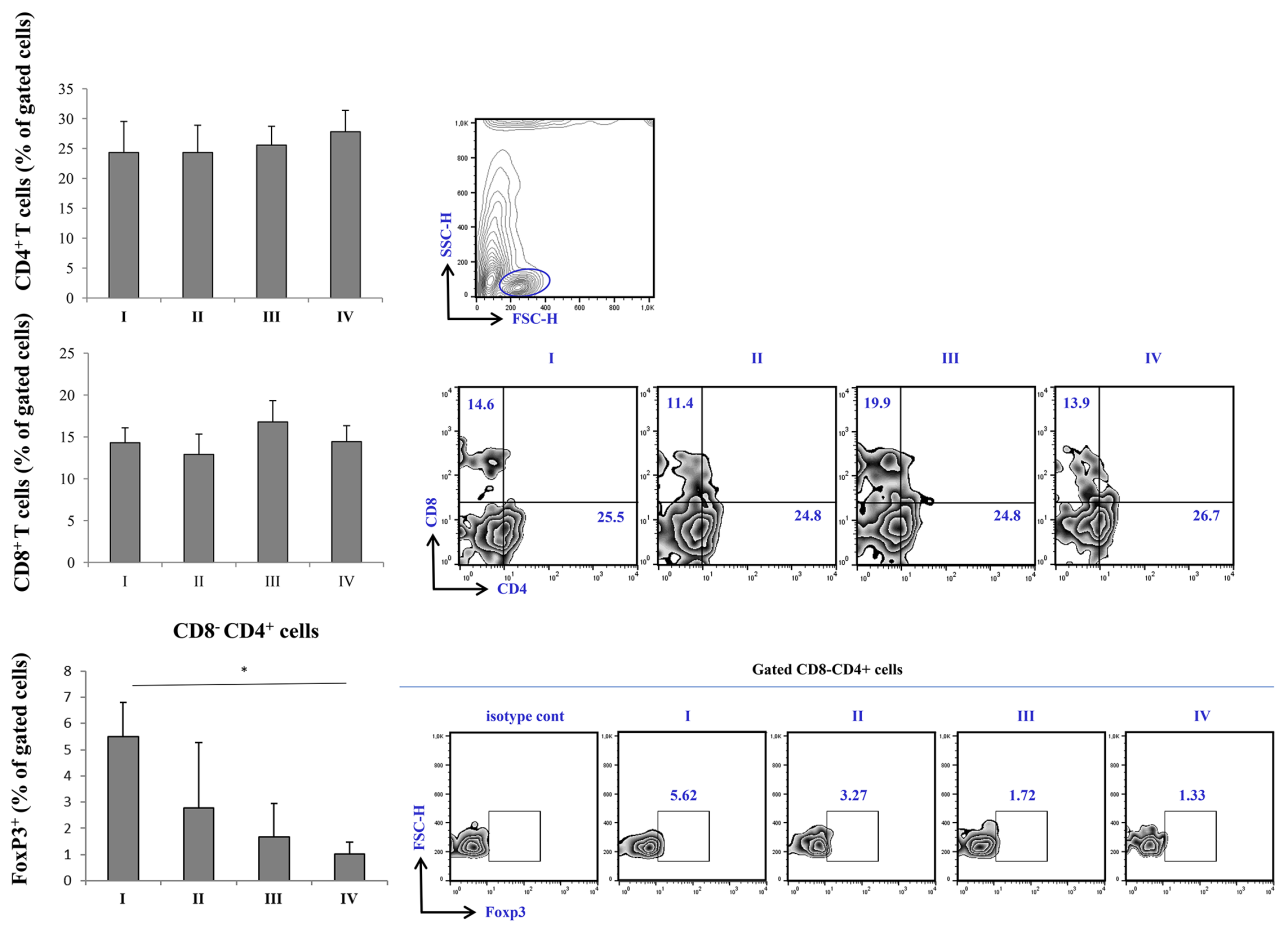
FACS analysis of T cell subpopulations in peripheral blood of COVID-19 patients is all progressive stages of disease. The graph and representative FACS plots displaying the percentage of CD8-CD4+ cells, CD4-CD8+ cells, CD8-CD4+ Fox3+ PBMCs detected in patients in all progressive stages of COVID-19. The Kruskal-Walli's test (with post-hoc Mann-Whitney U-test) was applied to evaluate statistically significant differences.

FACS analysis of T cell subsets revealed a trend of increasing percentage CD4^+^ T cells subsets with increasing disease severity, with the highest percentage being in stage IV of COVID-19. When it comes to CD8^+^ cells, similar trend was observed, however, the percentage of CD8^+^ was highest in stage III of the disease. Nonetheless, the percentage of CD8^+^ T cells in stage IV was higher when compared to stage I and II. The percentage of FoxP3^+^ cells was significantly lowered in stage IV in comparison to stage I of COVID-19 (Figure 3).

### Increased transcriptional activity of T cell subsets in severe COVID-19

**Figure 4:**
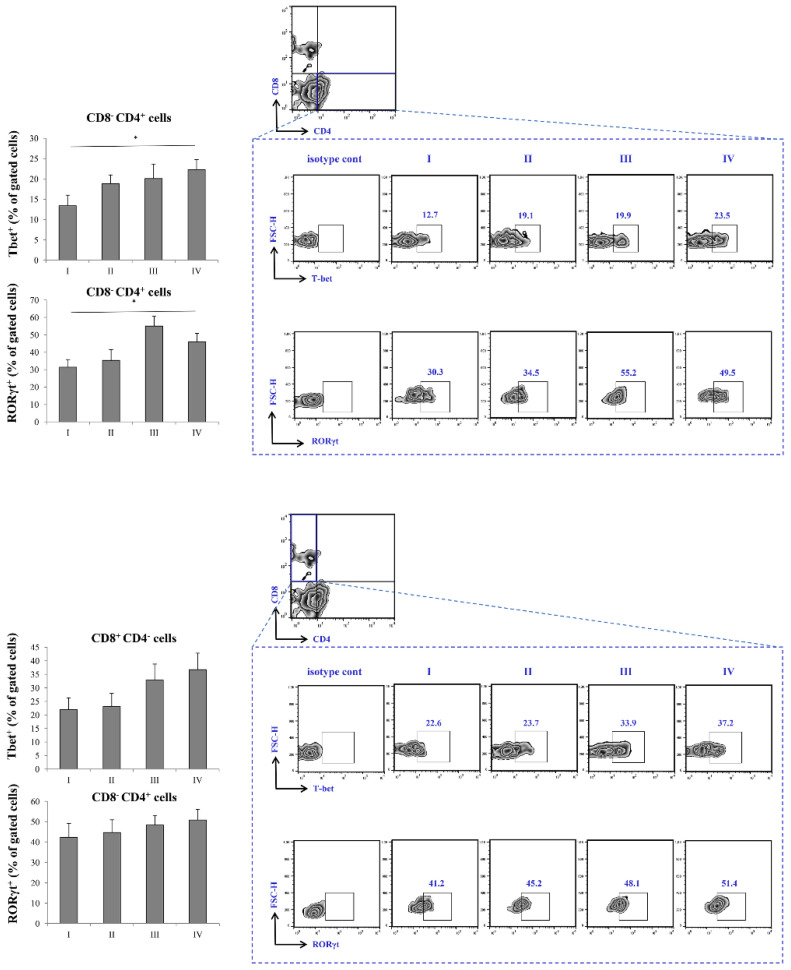
Increment in percentage of RoRγt+ and tBet+ Th cells in stage IV patients with COVID-19. The graph and representative FACS plots displaying the percentage of CD8-CD4+ RoRγt+ cells, CD8-CD4+ tBet+ cells, CD4-CD8+ RoRγt+ cells, CD4-CD8+ tBet+ cells, among PBMCs of patients in all progressive stages of COVID-19. The Kruskal-Walli's test (with post-hoc Mann-Whitney U-test) was applied to evaluate statistically significant differences.

**Figure 5:**
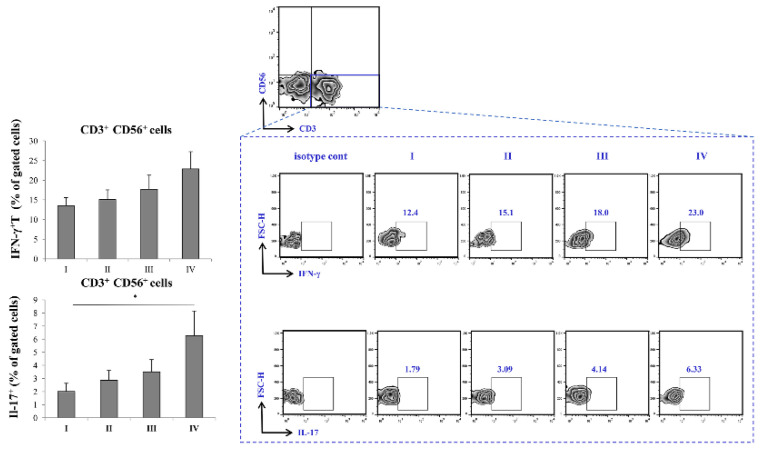
Increment in percentage of IFN-γ^+^ and IL-17^+^ T cells in stage IV patients with COVID-19. The graph and representative FACS plots displaying the percentage of CD56^-^CD3^+^ IFN-γ^+^ cells, CD56^-^CD3^+^ IL-17^+^ cells, among PBMCs of patients in all progressive stages of COVID-19. The Kruskal-Walli's test (with post-hoc Mann-Whitney U-test) was applied to evaluate statistically significant differences.

Expression of Tbet in CD8^-^CD4^+^ cells was highest in stage IV, with statistical significance in comparison to stage I. Similarly, the expression of RORγT was significantly higher in stage IV in comparison to stage I of the disease. When it comes to CD8^+^CD4^-^ T cells, the expression of Tbet was also highest in stage IV, however, the expression did not reach statistical significance. Similar trend was observed with expression of RORγT in CD8^+^CD4^-^ T cells, its percentage being highest in IV stage of COVID-19 (Figure 4).

### Augmented production of IFN-γ and IL-17 in severe COVID-19

The percentage of CD3^-^CD56^+^ IFN-γ producing cells was highest in stage IV of the disease, with trend of increment through stages I-IV. In addition, IL-17 was significantly elevated in stage IV of COVID-19 in comparison to stage I.

## Discussion

Immune response has been in the limelight of investigation since of the beginning of the pandemic, and it has been shown to shape the course and outcome of COVID-19 17. Until now, it is known that cytokine storm followed by acute respiratory failure is one of the main factors that contribute to COVID-19 severity 18. However, exact immune mechanisms, as well as exact cytokines involved in increment of COVID-19 severity are yet to be identified. COVID-19 has already been known to disrupt lymphocyte counts, especially T lymphocyte counts 19. In addition, it is highly likely that COVID-19 alters the ratio among T cell subsets 20. It has been also shown in numerous studies that SARS-CoV-2 induces immune dysfunction that could potentially lead to more severe disease and/or fatal outcome 21. In this study, we analyzed the systemic levels of cytokines produced by different T cell subsets of patients with different stages of COVID-19, in order to determine which T cell subset contributes to COVID-19 severity. All COVID-19 patients were divided into four groups, according to disease severity, as described previously. When it comes to the clinical and biochemical characteristics of COVID-19 patients, they fully monitor and reflect the progression of the stage of the disease (data not shown).

We analyzed cytokine levels of various cytokines in sera of COVID-19 patients through different stages of disease severity. The results showed significant increment of IFN-γ, IL-12, IL-17 and IL-23, and decrement in TGF-β in stage IV of COVID-19. These data are suggestive of intensified immune response in stage IV and are in line with previous studies that more severe or even fatal cases of COVID-19 might have amplified Th1 and Th17 immune response 22. Data regarding the role of TGF-β during COVID-19 are somewhat contradictory. Some authors agree that increment of TGF-β serum levels are indicative of better COVID-19 outcome, and results by others imply that higher levels of TGF-β are related to persistent long COVID-19 syndrome 23-25. On the other hand, some authors suggest that it is important to interpret the role of TGF-β in liaison to other cytokines, such as IFN-γ and IL-17 26. Our further analyses revealed moderate correlation between IL-12 and IFN-γ, IFN-γ and IL-17, as well as strong correlation between IL-23 and IFN-γ, IL-12 and IL-17. These results imply that, in severe cases of COVID-19, elevation of proinflammatory Th1 cytokines, especially IFN-γ and IL-12 are mostly followed by elevation of Th17 cytokines IL-17 and IL-23. These results are consistent with results of other studies that are suggestive of Th1/Th17 dysregulation during severe and/or critical COVID-19 27, 28. To support this hypothesis, it has been also shown that hyperactive Th1 or Th17 immune response could be an underlying mechanism of exacerbation of many other inflammatory diseases 29, 30. After having determined intercorrealtion between Th1 and Th17 cytokines during severe COVID-19, we further analyzed the correlation of IL-12, IFN-γ, IL-17 and IL-23 with clinical features and laboratory findings in severe COVID-19. Our results showed that IFN-γ was in strong positive association with age of severe COVID-19 patients. It has been emphasized before that advanced age is one of the risk factors for severe COVID-19 or its poor outcome 31. Interestingly, all the analyzed cytokines, IL-12, IL-23, IFN-γ and IL-17 were in strong positive association with CXR findings. This result is in line with observation that disruption and excessively active Th1 and Th17 immune response might lead to acute respiratory distress syndrome, which is also one of most common CXR findings in severe COVID-19 32, 33. IL-12 and IL-23 elevation was in moderate positive correlation with laboratory parameters such as urea, LDH, CK and PCT. It has been previously shown that elevation of LDH, CK and PCT is connected to worse COVID-19 outcome 34. On the other hand, all the analyzed cytokines were in negative association with markers of blood-gas exchange, such as pH, pO_2_, SaO_2_, and potassium levels, which denotes a more severe clinical presentation of COVID-19 upon elevation of Th1 and Th17 cytokines. ROC curves analysis revealed that IL-17, IFN-γ, IL-12 and IL-23 can be important markers for critical stage of COVID-19.

After outlining the clinical presentation of severe COVID-19 and its most notable laboratory findings, we analyzed whether there were any differences between T cell subsets in severe COVID-19. One of the first, and most prominent laboratory finding during COVID-19 is lymphopenia with altered ratio among T cell subsets, which could be accompanied with decreased levels of B, NK, NKT cells 35, 36. We found no significant difference in percentage of CD4^+^ and CD8^+^ T cells throughout different severity stages of COVID-19. We also analyzed CD3^+^CD56^+^ NKT like cells, as their role in inflammation could be closely related to function of T cells 37, 38. Similarly, FACS analysis did not retrieve significant difference throughout stages of COVID-19 severity when it comes to CD3^+^CD56^+^ NKT like cells (data not shown).

Analysis of regulatory T cells (Tregs) revealed decrement in percentage of CD4^+^ FoxP3^+^ T cells as well as in percentage of CD4^+^ IL-10 producing T cells (data not shown) in terminal stage of COVID-19. Regulatory T lymphocytes might be the source of IL-10. To support this presumption, when correlation analysis was preformed, we found strong positive correlation between percentage of CD4^+^IL-10^+^ cells and CD4^+^FoxP3^+^ cells in peripheral blood of COVID-19 patients in IV stage. Decrement in percentage of CD4^+^FoxP3^+^ Tregs in peripheral blood of COVID-19 patients in terminal stage of disease is in line with lower systemic TGF-β, one of the major products of Tregs.

Given the results above, as severe (stage IV) COVID-19 is outlined by increment of proinflammatory Th1 cytokines, IL-12 and IFN-γ, and Th17 cytokines, IL-17 and IL-23, we later on examined transcriptional activity of CD4^+^ and CD8^+^ T cells. Since Th1 response and secretion of IL-12 and IFN-γ are mostly governed by transcriptional factor Tbet, and Th17 response and secretion of IL-17 and IL-23 are mostly regulated by RORγT, we analyzed whether the expression of this transcriptional factor within T cells differs throughout progression of COVID-19 39, 40. When we analyzed CD8^+^ T lymphocytes, we found no significant difference in expression of either Tbet or RORγt during COVID-19 progression. Interestingly, when we analyzed CD4^+^ T helper lymphocytes, we found markedly increased expression of both, Tbet and RORγT in stage IV in comparison to stage I of COVID-19. Additionally, we found increased percentage of IFN-γ^+^ and IL-17^+^ CD56^-^CD3^+^ T cells in stage IV patients with COVID-19. Moderate correlation between percentages of CD4^+^RORgt^+^ and CD3^+^IL-17^+^ T cells in terminal stage of COVID-19 implicate that expression of RORγt transcriptional factor may induce production of IL-17 in T cells.

Presented results imply that the underlying mechanism behind increased COVID-19 severity might be overproduction of Th1 and Th17 cytokines by CD4^+^ Th cells. This It is consistent with earlier published data that CD4 cells, alongside with various immune cells, can be altered in more severe cases of COVID-19 35, 41-42. However, this is the first study to illustrate the exact mechanism preceding hyperinflamation and subsequent increased COVID-19 severity. We believe that increment in expression of transcriptional factors Tbet and RORγt leads to polarization of acquired cellular anti-SARS-CoV-2 immune response toward Th1 and Th17 and subsequent increment in production of Th1/Th17 cytokines IL-12, IFN-γ, IL-17 and IL-23, that facilitate inflammation and tissue destruction.

Increased transcriptional activity of CD4^+^ cells triggers activation of proinflammatory Th1 and Th17 immune response and, subsequent hyperproduction of proinflammatory IL-12, IFN-γ, IL-17, IL-23 affects the equilibrium of immune response that could potentially lead to resolution of the disease. Instead, hyperproduction of Th1 and Th17 proinflammatory cytokines and subsequent increment in the systemic concentration of cytokines leads to disruption of the immune system homeostasis and attenuated efficassy of immune system that innitiates the progression of COVID-19. Further studies are needed to investigate potential molecular cascade that leads to rapid dysregulation and harmful over-activation of proinflammatory immune response, so that new therapeutic strategies of impeding SARS-CoV-2 infection could be established.

## Figures and Tables

**Table 1:** Inter-ccorrelation between cytokines (IFN-γ, IL-23, IL-12, IL-17)

	IFN-γ		IL-23		IL-12		IL-17	
	Spearman's rho	p value	Spearman's rho	p value	Spearman's rho	p value	Spearman's rho	p value
**IFN-γ**	1.000		.317^**^	.000	.202^**^	.005	.179^*^	.014
**IL-23**	.317^**^	.000	1.000		.505^**^	.000	.238^**^	.001
**IL-12**	.202^**^	.005	.505^**^	.000	1.000		.074	.308
**IL-17**	.179^*^	.014	.238^**^	.001	.074	.308	1.000	

**. Correlation is significant at the 0.01 level (2-tailed). *. Correlation is significant at the 0.05 level (2-tailed).

**Table 2:** Correlation between percentages of CD4^+^RORgt^+^ and CD3^+^IL-17^+^ cells, and CD4^+^Foxp3^+^ and CD4^+^IL10^+^ cells.

	CD4^+^RORgt^+^		CD4^+^IL10^+^	
	Spearman's rho	p value	Spearman's rho	p value
**CD3^+^IL-17^+^**	.334**	.009	/	/
**CD4^+^Foxp3^+^**	/	/	.536**	.000

**. Correlation is significant at the 0.01 level (2-tailed). *. Correlation is significant at the 0.05 level (2-tailed).

**Table 3:** Correlation between Th1/Th17 cytokines and clinical parameters of COVID-19

Spearman's rho								
Clinical parameters	IL-17		IL-12		IL-23		IFN-γ	
	Correl. Coeff.	p value	Correl. Coeff.	p value	Correl. Coeff.	p value	Correl. Coeff.	p value
Feritin	-.041	.555	.075	.308	.132	.072	.092	.212
Age	-,122	,082	,089	,194	-,103	,135	,251^**^	,000
WB cell count	-,025	,726	,063	,362	-,096	,164	-,008	,904
Neutrophil %	,074	,298	,152^*^	,028	,102	,142	,035	,622
Lymphocyte %	-,041	,563	-,136	,050	-,043	,542	-,060	,394
Monocyte %	,062	,374	-,234^**^	,001	-,134	,055	-,002	,975
Eritrocite count	-,199^**^	,004	-,052	,454	-,008	,910	-,071	,308
Platelet count	-,040	,567	-,051	,461	-,044	,526	-,010	,889
Hemoglobin	-,015	,827	-,056	,418	-,071	,301	-,048	,490
Glucose	-,046	,509	,030	,662	-,030	,661	,037	,600
Urea	,058	,408	,188^**^	,006	,026	,703	,189^**^	,006
Creatinin	,104	,158	,122	,074	,062	,365	,110	,113
BILT	,015	,835	,172^*^	,018	,074	,307	,084	,255
BILD	-,051	,471	,229^**^	,001	,013	,854	,103	,158
AST	-,060	,393	,075	,285	,029	,676	,104	,136
ALT	-,008	,907	,026	,707	,031	,658	,037	,590
Albumins	-,045	,544	-,135	,051	-,008	,904	-,165^*^	,018
LDH	,015	,829	,161^*^	,027	,093	,204	,225^**^	,002
CK	,072	,303	,154^*^	,025	,205^**^	,003	,054	,436
CRP	-,044	,532	,224^**^	,001	,165^*^	,016	,036	,605
sat	-,144^*^	,041	-,354^**^	,000	-,149^*^	,030	-,227^**^	,001
pH	-,166^*^	,017	-,018	,794	-,087	,209	,097	,164
K	-,133	,057	,116	,090	-,004	,949	-,050	,474
Na	,013	,868	,165^*^	,016	,017	,806	,063	,366
Fe	,026	,731	-,214^**^	,005	-,104	,177	-,129	,094
CXR findings	,173^*^	,013	,469^**^	,000	,382^**^	,000	,378^**^	,000
Disease severity (groups I to IV)	,178^*^	,010	,463^**^	,000	,371^**^	,000	,360^**^	,000
D dimer	-,110	,115	,172^*^	,012	,050	,466	,095	,169
PCT	,049	,482	,199^**^	,003	,131	,057	,180^**^	,009
pO2	-,079	,258	-,369^**^	,000	-,226^**^	,001	-,269^**^	,000
pCO2	-,001	,988	,043	,535	-,039	,567	,027	,693
